# Genetic diversity, serotype, and antimicrobial profiles of *Riemerella anatipestifer* isolated from ducks and chickens in Thailand

**DOI:** 10.1016/j.psj.2026.106575

**Published:** 2026-02-02

**Authors:** Chutima Pathomchai-umporn, Sudtisa Laopiem, Kriangkrai Witoonsatian, Sittinee Kulprasetsri, Pun Panomwan, Manakorn Sukmak, Thaweesak Songserm, Worata Klinsawat, Nuananong Sinwat

**Affiliations:** a Department of Farm resources and production medicine, Faculty of Veterinary Medicine, Kasetsart University, Kamphaengsean campus, Nakorn Pathom, 73140 Thailand; b Department of Pathology, Faculty of Veterinary Medicine, Kasetsart University, Kamphaengsean campus, Nakorn Pathom, 73140 Thailand; c Conservation Ecology Program, School of Bioresources and Technology, King Mongkut’s University of Technology Thonburi, Bangkok 10150 Thailand

**Keywords:** Riemerella anatipestifer, Antimicrobial profiles, Whole-genome sequencing, Poultry

## Abstract

Riemerella anatipestifer, which causes New Duck Syndrome, poses a significant threat to poultry production, causesing respiratory distress, neurological signs, and septicemia primarily in ducks and occasionally chickens. *R. anatipestifer* outbreak control requires knowledge about their genetic diversity, serotypes, and antibiotic profiles, which are currently unavailable. In this study, R. anatipestifer was isolated from ducks and chickens in Thailand between 2021 and 2023 to characterize their genetic relatedness, antimicrobial profiles, and resistance genes. Seven different serotypes were identified in isolates from ducks. Non-typable strains were the most prevalent, followed by serotypes 7, 10, 1, 5, 11, and 17. However, only serotype 1 was identified in isolates from chickens. Antimicrobial susceptibility testing revealed high resistance to colistin and broad minimum inhibitory concentration (MIC) ranges for β-lactams, aminoglycosides, chloramphenicol, sulfamethoxazole, and trimethoprim. Of the 17 representative isolates analyzed by WGS, the most prevalent resistance genes were *tet*(X2) and *lnu*(I). Phylogenetic analysis based on core-genome single nucleotide polymorphisms (SNPs) categorized the isolates into four main clusters. Most of Thai isolates from both ducks and chickens clustered together, indicating the circulation of endemic strains within the region. This is the first comprehensive study of R. anatipestifer isolated from ducks and chickens in Thailand. This research illustrates the value of enhancing basic biosecurity and movement control among farms. The data also provide a valuable foundation for the development of antibiotic use guidelines and vaccines, which will enhance the judicious and minimal use of antibiotics in poultry production.

## Introduction

*Riemerella anatipestifer* is a gram-negative, rod-shaped bacterium from the family *Flavobacteriaceae*. It is the causative agent of New Duck Syndrome, a highly contagious disease primarily affecting ducks (Li et al., 2011; Ruiz & Sandhu, 2013). Mortality rates from *R. anatipestifer* infections can vary between 5% and 75%, but morbidity rates can reach 100% (Yu et al., 2008). Young ducks, particularly those less than 8 weeks of age, are highly susceptible. Significant mortality can occur within 1–2 days following the onset of symptoms (Ruiz & Sandhu, 2013). However, *R. anatipestifer* can also infect chickens, causing respiratory distress, diarrhea, and stunted growth (Li et al., 2011; Tzora et al., 2021).

*R. anatipestifer* poses a considerable economic risk to the poultry sector, especially in Asia, with China, Vietnam, Malaysia, and Thailand leading in duck production, respectively, in order of scale (FAO, 2022). The growing demand for poultry production in Thailand has increased the adoption of intensive poultry farming systems, as evidenced by the rising export value of meat duck products, which increased to approximately 955.35 million Thai baht from 2022 to 2023 (Statista, 2025). Thus, to effectively control *R. anatipestifer* outbreaks, a comprehensive understanding of its epidemiology and geographic distribution must be established.

In addition to epidemiological studies, prudent use of antibiotics, especially beta-lactams and aminoglycosides, has been essential in reducing disease severity (Lutful Kabir, 2010). The increasing prevalence of antimicrobial resistance (AMR) in *R. anatipestifer*, particularly within the poultry farming sector, has become a significant veterinary concern (Liu et al., 2024). Thus, antibiotics considered critically important for human health must be preserved (WHO, 2024) and their restricted use in animals must be emphasized to mitigate the emergence of more highly resistant strains. However, the distribution, genetic relatedness and underlying mechanisms of AMR in *R. anatipestifer* remains largely unexplored.

Whole-genome sequencing (WGS) offers promising molecular approaches to obtain a deeper understanding of bacterial characteristics associated with their genetic relatedness, antimicrobial resistance, and virulence genes. Furthermore, WGS provides a comprehensive view of the genomic landscape, allowing the discovery of novel antimicrobial resistance genes (ARGs) and the elucidation of resistance mechanisms.

Although *R. anatipestifer* infection is a significant duck pathogen, it also infects chickens. Nonetheless, its prevalence and genetic characteristics in both species remain poorly understood. This is the first study in Thailand to characterize the antimicrobial profiles, serotypes, and genetic relatedness of *R. anatipestifer* isolates from Thai ducks and chickens. The findings of this study provide valuable insights into strain diversity and antibiotic resistance genes in *R. anatipestifer*, which are crucial for improving disease management and antimicrobial stewardship in Thailand’s poultry industry.

## Materials and methods

### *R. anatipestifer* strains

In total, 47 *R. anatipestifer* field strains were isolated from diseased birds between 2021 and 2023 and maintained in our laboratory collection for this study. Of these, 37 isolates were isolated from ducks exhibiting respiratory distress and neurological signs (rolling, paddling, or tremors) from 23 farms, while 10 isolates were isolated from chickens exhibiting depression and respiratory signs from 2 farms. A convenience sampling method was employed for sample collection. Visceral organs with fibrinopurulent polyserositis lesions were aseptically collected from the affected birds and immediately sent to the microbiology laboratory for bacterial culture within 1 hour of sample collection. Necropsy examination and bacterial isolation and identification were performed at Kamphaeng Saen Veterinary Diagnostic Center (KVDC), the Faculty of Veterinary Medicine, Kasetsart University, Kamphaeng Saen Campus, Thailand. All samples were cultured on tryptic soy agar (Difco™ Laboratories, New Jersey, USA) containing 5% sheep blood and incubated at 37°C under microaerophilic conditions (5% CO_2_). Presumptive *R. anatipestifer* colonies were identified based on their morphology, Gram staining, and biochemical characteristics. 16SrRNA gene sequencing was performed to confirm the identity of isolates. Polymerase chain reaction (PCR) amplification was conducted using specific primers, as described previously (Tsai et al., 2005). The positive PCR amplicons were submitted to U2bio (Seoul, South Korea) for nucleotide sequencing. Sequence data were compared with those on the GenBank database using the Basic Local Alignment Search Tool software. Confirmed pure colonies were stored in CRYOBANK™ (Mast Group, Liverpool, United Kingdom) and stored at −80°C until further analysis.

### Serotyping

All *R. anatipestifer* strains were serotyped through the agar gel precipitin test using 20 specific antisera (antisera 1–21, excluding antiserum 4) obtained from the National Institute of Animal Health, Department of Livestock Development, Thailand. Positive serotyping is indicated by the formation of a visible precipitin line between the antigen and antiserum wells. Results with no precipitin line were designated as non-typable strains.

### Antimicrobial susceptibility testing

The antimicrobial susceptibility of *R. anatipestifer* isolates was determined using the agar dilution method, in accordance with the Clinical and Laboratory Standards Institute guidelines (CLSI, 2018). *Escherichia coli* ATCC® 25922 and *Pseudomonas aeruginosa* ATCC® 27853 were used as quality control reference strains. The 11 antibiotics tested in this study were amoxicillin, ampicillin, ceftriaxone, gentamicin, streptomycin, doxycycline, tetracycline, chloramphenicol, colistin, sulfamethoxazole, and trimethoprim (Sigma-Aldrich®, USA). Antimicrobial concentrations ranged from 0.03 to 128 µg/mL (Supplementary Table 1), as determined using previously established protocols (Sun et al., 2012).

### Genomic DNA preparation, genome assembly and quality assessment

The representative Seventeen isolates of *R. anatipestifer* were obtained from ducks and chickens. The isolates derived from ducks were primarily selected based on their distinct serotypes; however, the most common serotypes, including serotype 7 and non-typable strains, were also randomly chosen from different farm areas. The chicken isolates, identified as serotype 1 and originating from the same farm, were selected based on different bird ages and sampling times throughout 2022 (Supplementary Table 1). Genomic DNA was extracted from the 17 *R. anatipestifer* isolates using the TIANamp Bacteria DNA Kit (Tiangen, Beijing, China) according to the manufacturer’s protocol. The DNA samples were submitted to Novogene Co., Ltd. (Beijing, China) for library preparation and next-generation sequencing. Sequencing libraries were constructed using the Rapid Plus DNA LibPrep Kit for Illumina V2 (ABclonal Technology, MA, USA). The libraries were subsequently sequenced on the Illumina NovaSeq X Plus platform, generating 150 bp paired end reads. Genome assembly and downstream bioinformatic analyses were performed using the Galaxy platform (https://usegalaxy.org/). Initial raw read quality was evaluated with FastQC (version 0.74+galaxy1) (Andrews, n.d.). Quality filtering and adapter trimming were conducted using fastp (version 0.24.0+galaxy4) (Chen et al., 2018) with a minimum Phred quality score threshold of 20. *De novo* assembly was performed using Unicycler (version 0.5.1+galaxy0) (Wick et al., 2017), and contigs shorter than 200 bp were excluded from the final assembly. Assembly metrics were assessed using QUAST (version 5.3.0+galaxy0) (Mikheenko et al., 2018), while genome completeness and contamination were verified using CheckM (version 1.2.3+galaxy0) (Parks et al., 2015).

### Phylogenetic analysis and characterization of antibiotic resistance genes (ARGs) and virulence genes (VRGs)

A core single nucleotide polymorphism (SNP)-based phylogenetic tree was constructed to investigate the evolutionary relationships among the isolates. The *R. anatipestifer* ATCC 11845 genome (GenBank accession: NC_014738.1) served as the reference. SNP calling was performed on the quality-filtered reads of the 17 isolates, along with 20 additional *R. anatipestifer* genomes retrieved from the NCBI GenBank database, using Snippy (version 4.6.0+galaxy0) (Seemann, 2015). The identified SNPs were subsequently concatenated into a core SNP alignment using Snippy-core. A Maximum Likelihood (ML) phylogenetic tree was then constructed using IQ-TREE (version 2.4.0+galaxy1) (Nguyen et al., 2014) with 1,000 bootstrap replicates to assess nodal support. The final phylogenetic tree was visualized and annotated using the Interactive Tree Of Life (iTOL) v6 (Letunic & Bork, 2024).

Antimicrobial resistance and virulence genotypes were identified from the assembled contigs using AMRFinderPlus (version 3.12.8+galaxy0) (Feldgarden et al., 2021). To ensure high-confidence identification, a minimum coverage and identity threshold of 95% was applied against the NCBI database. To infer the genomic location (plasmid-borne vs. chromosomal) of the identified AMR genes, a sequencing depth-based approach was employed. The quality-filtered reads were re-mapped to the assembled contigs using Bowtie2 (version 2.5.3+galaxy1) (Langmead et al., 2009). The mean depth of coverage for each contig was calculated using Samtools coverage (version 1.22+galaxy3) (Danecek et al., 2021). The baseline chromosomal coverage was established using the median depth of all contigs across the entire genome to provide a robust reference and minimize the influence of high-copy elements or repeat regions. The relative Copy Number (CN) for each AMR-harboring contig was determined using the following formula:

"Copy Number " ("CN")"= " "Mean Depth pf Target Contig" /"Median Depth of Genome"

Contigs exhibiting a CN approximately equal to 1.0 were interpreted as chromosomal, while those with a CN significantly higher than the genomic median (e.g., > 1.5–2.0) were considered putative plasmid-borne or located on multi-copy mobile genetic elements.

## Results

### *R. anatipestifer* serotypes of isolates from ducks and chickens

In total, 47 *R. anatipestifer* isolates were serotyped (Table 1). Seven different serotypes were identified in the isolates from ducks, with non-typable strains being the most prevalent, followed by serotypes 7, 10, 1, 5, 11, and 17. In contrast, only serotype 1 was detected in isolates from chickens.

**Table 1:** Differences in *Riemerella anatipestifer* serotypes isolated from ducks and chickens (n = 47).

Year of sample collection	Serotype no. (number of isolates)	
	Duck (n = 37)	Chicken (n = 10)
2021	7 (5) 11 (1)Non- typeable (5)	N/A
2022	1 (1) 5 (2) 11 (1)Non- typeable (11)	1 (10)
2023	1 (1) 7 (2) 10 (4) 17 (1)Non- typeable (3)	N/A
Total	37	10

### Antimicrobial susceptibility profiles

Minimum inhibitory concentration (MIC) testing of the 47 isolates revealed varying degrees of susceptibility to 11 antimicrobial agents (Table 2). A comparison of MIC values between the isolates from ducks and chickens is presented in Table 3. Amoxicillin exhibited an MIC for inhibiting 50% growth (MIC_50_) of 4 µg/mL and an MIC_90_ of 32 µg/mL, while ampicillin showed an MIC_50_ of 2 µg/mL and MIC_90_ of 32 µg/mL. Ceftriaxone demonstrated even lower values with MIC_50_ ≤ 0.03 µg/mL and MIC_90_ of 0.125 µg/mL. Gentamicin and streptomycin exhibited the same MIC_50_ and MIC_90_ values at 32 µg/mL and 64 µg/mL, respectively. Doxycycline achieved MIC_50_ and MIC_90_ values of 1 and 4 µg/mL, respectively, while tetracycline showed 8 and 16 µg/mL, respectively. Chloramphenicol exhibited MIC_50_ and MIC_90_ values of 2 and 16 µg/mL, respectively. Colistin and sulfamethoxazole demonstrated identical MIC_50_ and MIC_90_ values both ≥128 µg/mL. Trimethoprim achieved MIC_50_ and MIC_90_ of 32 and ≥128 µg/mL, respectively.

**Table 2:** Minimum inhibitory concentrations of antimicrobial agents against *Riemerella anatipestifer* isolates (n = 47).

Antimicrobial agent	Test range (µg/mL)	No. of isolates with MIC (µg/mL)													MIC_50_ (µg/mL)	MIC_90_ µg/mL)
		≤0.03	0.06	0.125	0.25	0.5	1	2	4	8	16	32	64	≥128		
Amoxycillin	0.25–128					3		1	24	7	7	4	1		4	32
Ampicillin	0.25–128					3	10	12	6	3		11	1	1	2	32
Ceftriaxone	0.03–128	26	14	3	2	1			1						≤0.03	0.125
Gentamicin	0.03–128	2	2				1	1	3	2	2	13	20	1	32	64
Streptomycin	0.25–128								1		11	23	8	4	32	64
Doxycycline	0.06–128				1	3	20	16	4	2	1				1	4
Tetracycline	0.03–128	2					1	10	10	18	1				8	16
Chloramphenicol	0.25–128					1		39	1	1	1	2		2	2	16
Colistin	0.12–128													47	≥128	≥128
Sulfamethoxazole	0.06–128		3								2	2		40	≥128	≥128
Trimethoprim	0.06–128					2		1	1	4	8	10	3	18	32	≥128

**Table 3:** MIC_50_ and MIC_90_ of antimicrobial agents against *Riemerella anatipestifer* isolates.

Antimicrobial agent	Test range (µg/mL)	Ducks (n = 37)		Chickens (n = 10)	
		MIC_50_ (µg/mL)	MIC_90_ µg/mL)	MIC_50_ (µg/mL)	MIC_90_ µg/mL)
Amoxycillin	0.25–128	4	16	16	32
Ampicillin	0.25–128	2	32	2	32
Ceftriaxone	0.03–128	0.03	0.25	0.03	0.06
Gentamicin	0.03–128	32	64	64	64
Streptomycin	0.25–128	32	64	32	64
Doxycycline	0.06–128	2	4	1	2
Tetracycline	0.03–128	4	16	4	8
Chloramphenicol	0.25–128	2	8	2	32
Colistin	0.12–128	≥128	≥128	≥128	≥128
Sulfamethoxazole	0.06–128	≥128	≥128	≥128	≥128
Trimethoprim	0.06–128	32	≥128	32	≥128

### SNP-based phylogenetic clustering of *R. anatipestifer*

**Fig. 1:**
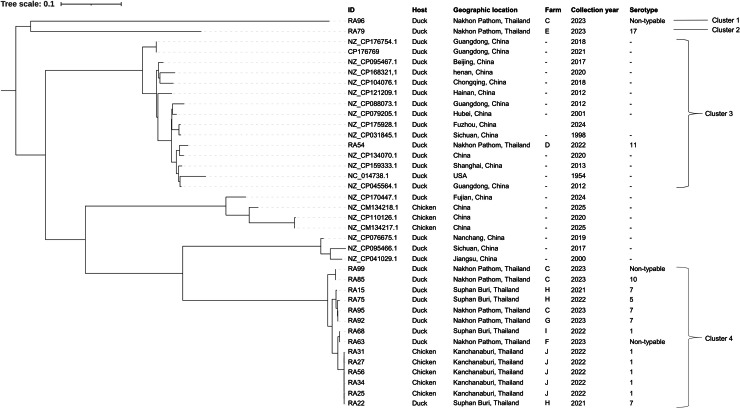
A core single nucleotide polymorphism (SNP)-based phylogenetic tree revealed the genetic relatedness of 17 representative *Riemerella anatipestifer* strains through cluster analysis.

A phylogenetic tree was constructed based on core-genome single nucleotide polymorphisms (SNPs) from the 17 *R. anatipestifer* isolates. Except for isolate RA54, most isolates were phylogenetically distinct from the NCBI GenBank reference strains. The isolates were categorized into four main clusters. Cluster 1 consisted of a single non-typable isolate (RA98) from a duck, while Cluster 2 contained serotype 17 (RA79). Cluster 3 comprised only serotype 11 (RA54), which exhibited close genetic relatedness to Chinese variants. The remaining fourteen isolates, representing serotypes 1, 5, 7, 10, and non-typable strains, were grouped within Cluster 4 (Fig. 1).

### Characterization of ARGs and VRGs

All 17 isolates examined using WGS contained at least of one antibiotic resistance gene (ARG). The aminoglycoside resistance genes *aadS* was identified in only one sample. Tetracycline resistance was observed with *tet*(X2) in 94.1% (16/17) of isolates and *tet*(Q) in 5.9% (1/17) of isolates. Lincosamide resistance was predominantly carried out by *lnu*(I) (14/17, 82.4%), while *lnu*(AN2) was detected in only one isolate. Among macrolide resistance genes, *ere*(D) was the most frequently detected in eight isolates (47.1%), whereas *erm*(F), *mef*(En2), and *estT* were each identified in a single isolate. Beta-lactamase genes *bla*_RASA-1_ and *bla*_RAD-1_ were found in 35.3% (7/17) and 5.9% (1/17) of isolates, respectively. Notably, no virulence-related genes (VRGs) were detected in any of the isolates.

We performed an additional analysis of antibiotic resistance gene copy numbers (CN) to predict their genomic localization, differentiating between chromosomal and plasmid-associated determinants. The quantitative analysis of 17 isolates demonstrated a varied resistome landscape, with CN values between 0.71 and 2.95 (Supplementary Table 2). Although most isolates preserved a chromosomal baseline (approximately 1.0), some isolates demonstrated notable gene amplification. Isolate number RA54 showed the highest copy number in this study, with *bla*_RASA-1_ reaching a copy number of 2.95. The increased copy number might be linked with increased phenotypic resistance, as revealed by the higher ceftriaxone MIC levels in Table 3. Meanwhile, isolate RA85 exhibited a notable amplification of *tet*(X2) with a copy number of 2.78, but its MIC values for tetracycline and doxycycline were comparable to those of isolates containing only a single chromosomal copy of the gene.

### The correlation between antimicrobial resistance phenotypes and genotypes

The correlation between MIC phenotypes and their corresponding ARG profiles is summarized in Table 3. Among the beta-lactams, 41.2% of isolates harbored beta-lactamase genes, specifically *bla*_RASA-1_ or a combination of *bla*_RASA-1_ and *bla*_RAD-1_. Notably, 58.8% of isolates demonstrated different MIC values for beta-lactam despite the absence of known corresponding genes. For aminoglycosides, only one isolate (5.9%) carried the *aadS* gene. Despite the absence of genetic markers, 94.1% of isolates demonstrated significant phenotypic resistance to gentamicin and streptomycin, with MIC values reaching to 128 µg/mL. In contrast, tetracycline resistance showed high genotypic correlation. Fifteen isolates (88.2%) carried *tet*(X2), while one isolate harbored a combination of *tet*(X2) and *tet*(Q). These genes corresponded with MICs for tetracycline and doxycycline ranging up to 16 ug/mL. Other antibiotics, including chloramphenicol, colistin, sulfamethoxazole, and trimethoprim, were also assessed, but no associated ARGs were detected via WGS. Furthermore, because the MICs for macrolides and lincosamides were not tested, the genotype-phenotype correlation for these classes remains unexamined. Table 4

**Table 4:** Distribution of antibiotic resistance genes and their correlation with MIC values in *R. anatipestifer isolates* (n=17).

Resistance genes pattern	No. of isolates (%)	Antibiotic resistance	MIC range (n) (ug/mL)
**Beta-lactam Genes**			
*bla*_RASA1_	6 (35.3)	AMP	2(2), 4(2), 8(1), 32(1)
		AMX	4(5), 16(1)
		CEF	0.03(2), 0.06(4)
*bla*_RASA1_-*bla*_RAD-1_	1 (5.9)	AMP	1
		AMX	4
		CEF	0.25
no corresponding genes	10 (58.8)	AMP	0.5(1), 1(1), 2(4), 4(1), 32(3)
		AMX	0.5(1), 2(1), 4(3), 8(1), 16(1), 32(2), 64(1)
		CEF	0.03(7), 0.06(5)
**Aminoglycosides**			
*aadS*	1 (5.9)	GEN	32
		STR	32
no corresponding genes	16 (94.1)	GEN	1(1). 4(1), 8(1), 16(1), 32(6), 64(5), 128(1)
		STR	4(1), 16(7), 32(6), 64(1), 128(1)
**Tetracycline Genes**			
*tet(X2)*	15 (88.2)	TET	1(1), 2(5), 4(3), 8(3), 16(3)
		DOX	0.25(1), 0.5(1), 1(7), 2(5), 16(1)
*tet(X2)*-*tet(Q)*	1 (5.9)	TET	4
		DOX	1
no corresponding genes	1 (5.9)	TET	8
		DOX	2

## Discussion

This study characterized 47 *R. anatipestifer* isolates obtained from clinically diseased ducks and chickens raised in the central region of Thailand. Six different serotypes (1, 5, 7, 10, 11, and 17) were detected in duck samples, indicating regional serotype variation, as demonstrated in previous studies. Serotypes 1, 2, 6, and 7 were identified in Shandong, China (DLD, 2023; Lyu et al., 2023), whereas serotypes B, 3, 11, and 8 were predominantly found in *R. anatipestifer* specimens isolated from ducks in Taiwan (Chang et al., 2019). A study conducted in Australia identified serotypes 1, 6, and 8 as the predominant serotypes (Omaleki et al., 2021). A previous study in Thailand conducted between 1994 and 1999 identified serotypes 7, 5, 10, 21, and 1 in strains isolated from ducks (Pathanasophon et al., 2002). Variations in serotype distribution across regions and potentially over time within the same location emphasizes the need for continuous surveillance to monitor trends and inform region-specific control strategies, such as targeted vaccination programs and updated poultry farm biosecurity measures.

Our study identified *R. anatipestifer* serotype 1 in chickens. While clinical *R. anatipestifer* infections are less commonly reported in chickens than in ducks, an outbreak in Greek broilers was previously recorded (Tzora et al., 2021), with respiratory distress and fibrinous polyserositis confirming clinical disease in the tested chickens. However, diagnosing *R. anatipestifer* in chickens is challenging because the clinical signs and gross lesions often resemble those caused by other bacterial pathogens, such as *Escherichia coli* and *Ornithobacterium rhinotracheale* (Ruiz & Sandhu, 2013). Furthermore, the pathogen’s microaerophilic growth requirements can hinder its detection in routine laboratory procedures, potentially resulting in underdiagnosis (Tzora et al., 2021).

In this study, we found a high number of non-typeable isolates. This may be due to novel serotypes or antigenic changes among existing serotypes, which the current serotyping scheme may be unable to sufficiently classify, given the substantial time requirement of the test. Improved serotyping methods, such as WGS or other molecular methods, are needed to sufficiently establish *R. anatipestifer* diversity and augment epidemiological studies.

The analysis of SNP-based phylogenetic clustering of *R. anatipestifer* isolates, in comparison with *R. anatipestifer* genomes from the NCBI GenBank database, discovered that the majority of isolates, originating from various regions of China, were categorized into four clusters (1, 2, 3, and 4). Clusters 1, 2, and 4 indicated that the isolates from this study were distinct from those originating in China; meanwhile, only one sample (serotype 11), derived from a duck, clustered with the reference strains. The findings suggest that the *R. anatipestifer* strains present in Thai poultry farms, including ducks and chickens may be endemic to the area, resulting in continuous circulation and recurrent outbreaks. Our phylogenetic data highlight the crucial importance of strict biosecurity practices among poultry farms, especially in Thailand's Central region, due to its intensive poultry production (DLD, 2023). Multiple factors, including unrestricted human and animal movement, inadequate transportation controls across areas, and insufficient cleaning and disinfection during downtime periods, significantly contribute to the spread and circulated of *R. anatipestifer* strains among farms.

Notably, a single isolate (serotype 11) in this study demonstrated a close evolutionary relationship with strains from China, suggesting either a shared ancestry or a recent common origin. This finding emphasized the potential for the cross-border transmission of *R. anatipestifer*. Consequently, in cases of importing breeders from different countries, monitoring programs should be implemented to mitigate the risk of introducing new strains into Thai poultry farms. However, this study is limited due to its small sample size. Analyzing a larger number of isolates in further studies could provide a more comprehensive understanding of the evolutionary linkages and genetic diversity of the strains.

The present study evaluated the antimicrobial susceptibility of 47 *R. anatipestifer* isolates against 11 commonly used antibiotics. Regional variations in MIC values were observed that were not reported previously. Beta-lactams (amoxicillin and ampicillin) exhibited higher MIC values, potentially reflecting the extensive use of these antibiotics in Thai livestock (Lekagul et al., 2023). Conversely, cephalosporin MIC values were lower than those reported in Chinese mainland and Taiwan (Chang et al., 2003; Sun et al., 2012), possibly due to stricter regulations on veterinary cephalosporin use in Thailand (Lekagul et al., 2023; WHO, 2024). Similarly, aminoglycosides showed lower MIC values than those reported in Poland, China, and Taiwan (Chang et al., 2003; Nowaczek et al., 2023; Sun et al., 2012), indicating varying antimicrobial pressures across regions. Tetracycline susceptibility also varied, with lower MICs for doxycycline but higher for tetracycline than those reported in Chinese mainland and Taiwan (Chang et al., 2003; Han et al., 2024; Sun et al., 2012). Despite the ban on chloramphenicol in food-producing animals in Thailand (Hanekamp & Bast, 2015; MOPH, 2020), some isolates still showed resistance to this antibiotic, emphasizing the importance of continuous monitoring for potential emergence of resistance. High MICs were also observed for sulfonamides (sulfamethoxazole), consistent with findings in other countries (Chang et al., 2003; Sun et al., 2012). The combination of sulfamethoxazole and trimethoprim exhibited synergistic effects in other studies (Nowaczek et al., 2023; Sun et al., 2012), indicating its therapeutic potential despite the observed resistance to individual components. Notably, all isolates were highly resistant to colistin, with MIC values > 128 µg/mL, a finding consistent with a recent report from Poland (Nowaczek et al., 2023).

The present study performed WGS to investigate the presence of ARGs in representative of *R. anatipestifer* isolates. Several clinically relevant ARGs were identified, including genes conferring resistance to beta-lactams (*bla*_RASA-1_, *bla*_RAD-1_), tetracyclines (*tet*(X), *tet*(Q)), macrolides (*erm*(F), *ere*(D),*mef*(En2) and *estT*) and lincosamide (*Inu*(I), *Inu*(AN2)). In this study, most isolates exhibited a chromosomal baseline with copy numbers of approximately 1.0. These findings are consistent with previous studies (Jiang et al., 2023; Li et al., 2022; Yang et al., 2024) suggesting that ARGs in *R.anatipestifer* might be predominantly mediated by chromosomes, while plasmid-mediated resistance genes do not serve as a primary mechanism for the dissemination of various ARGs within *R. anatipestifer* populations.

Recent reports indicate the presence of the chromosome-encoded enzymes *bla*_RASA-1_ (a Class A extended-spectrum beta-lactamase) and *bla*_RAD-1_ (a Class D carbapenemase) (Li et al., 2023; Luo, Li, et al., 2022) in *R.anatipestifer* isolates from ducks and geese in China (Liu et al., 2024). Vertical transmission ensures their stable persistence and dissemination among offspring due to their characteristic localization on chromosomes. Nonetheless, continuous monitoring of their development is necessary. For instance, a previous report found *bla*_RASA-1_, a beta-lactamase gene that is typically chromosomal but has been discovered integrated within a potential transposon flanked by IS982 elements (Luo et al., 2022). This genetic composition indicates a high potential for horizontal gene transfer (HGT) of the *bla* gene family among and between poultry populations, representing a considerable risk to animal health and public safety.

The *tet*(X) gene confers resistance to tetracyclines and tigecycline, a critical last-resort antibiotic in medicine for humans. These genes and their variants can be either chromosomally encoded or carried on plasmids, promoting their spread. Most isolates in this study had *tet*(X2). This differs from findings in China and Poland (Li et al., 2022; Nowaczek et al., 2023; Xihui et al., 2023) found different variant *tet*(X), which identified different tet(X) variants in various regions.

Most of the isolates present *ere*(D), which is particularly resistant to erythromycin. Macrolides are an important class of antibiotics used in both human and veterinary medicine. The identification of *ere*(D) in this study is consistent with previous findings in China, where multiple macrolide and other resistance genes were detected (Zhu et al., 2022) The finding of *estT*, which hydrolyzes 16-membered macrolides often used in livestock production, like tylosin, is notably interesting in this study. In Thailand's poultry farm industry, tylosin is mainly used for the treatment of Mycoplasma in chickens, with rarely use in ducks. This result suggests that *R.anatipestifer* may serve as a reservoir for this resistance genes, as the usage of other antibiotics creates selective pressure.

The emergence of *lnu*(I) in *R.anatipestifer*, a novel gene that confers resistance to lincosamides, has recently been documented by (Yang et al., 2024). In this study, we detected this gene in 82.4% of our isolates. While lincosamides are typically used for treating Gram-positive bacterial infections, *R. anatipestifer*, which is a Gram-negative bacterium, has demonstrated the presence of *lnu*(I), and this gene can be associated with mobile genetic elements (MGEs), including integrative and conjugative elements (ICEs) and insertion sequences (IS), which facilitate its horizontal movement among many hosts and environmental settings.

The correlation between Minimum Inhibitory Concentration (MIC) values and the presence of corresponding antimicrobial resistance (AMR) genes was examined. Among the beta-lactams, isolates containing *bla*_RASA-1_and *bla*_RAD-1_exhibited a wide range of MIC values. The lack of beta-lactamase genes in 58.8% of the isolates indicates that these phenotypes may be mediated by non-enzymatic mechanisms. This resistance may result from other resistance mechanisms such as mutations in penicillin-binding proteins (PBPs) or alterations in outer membrane porins (Galgano et al., 2025). Additionally, the other antibiotics tested in this study, such aminoglycosides, colistin, and chloramphenicol, exhibited high MICs without corresponding resistance genes, indicating the potential presence of undiscovered or novel resistance genes contributing to these resistances. Consequently, future study involving larger sample sizes is warranted to investigate the presence of novel or less common resistance mechanisms.

Overall, this study is the first to describe the complex epidemiology of *R. anatipestifer*, as characterized by serotype distribution and the emergence of AMR. The detection of various ARGs, including those conferring resistance to critically important antimicrobials, underscores the critical need for continuous AMR surveillance and monitoring in *R. anatipestifer*. Improved diagnostic techniques, such as WGS, are crucial for accurate identification of emerging strains and resistance profile characterization. Furthermore, responsible antimicrobial stewardship programs, including thoughtful antibiotic use, improved biosecurity practices, and vaccination strategies, are essential for effective management and control of both *R. anatipestifer* infections and AMR spread in poultry.

## Ethics approval

The KASETSART UNIVERSITY Institutional Animal Care and Use Committee (ACKU66-VET-072) approved all procedures for sample collection in this study. These procedures were also found to be in accordance with the animal care and use guidelines established by the Ethical Review Board of the Office of National Research Council of Thailand (NRCT) for scientific research. The committee granted permission for the animal care and use as detailed in the research study and animal use protocol.

## Funding

This study was financially supported by the Faculty of Veterinary Medicine, Kasetsart University and partially funded by the National Research Council of Thailand (NRCT) under Project ID N42A660897

## CRediT authorship contribution statement

**Chutima Pathomchai-umporn:** Writing – original draft, Investigation, Formal analysis, Data curation. **Sudtisa Laopiem:** Investigation. **Kriangkrai Witoonsatian:** Writing – review & editing, Investigation. **Sittinee Kulprasetsri:** Writing – review & editing, Investigation. **Pun Panomwan:** Investigation. **Manakorn Sukmak:** Investigation. **Thaweesak Songserm:** Methodology, Investigation. **Worata Klinsawat:** Investigation. **Nuananong Sinwat:** Writing – review & editing, Writing – original draft, Methodology, Investigation, Conceptualization.

## Disclosures

The authors declare that they have no known competing financial interests or personal relationships that could have appeared to influence the work reported in this paper.

## Appendix A. Supporting information

All data supporting the findings are provided within the manuscript.

## Appendix B Supplementary materials

Image, application 1

Image, application 2
