# Supplementary material for: Genetic diversity, serotype, and antimicrobial profiles of Riemerella anatipestifer isolated from ducks and chickens in Thailand
Source: Poult Sci. 2026 Feb 2;105(4):106575. doi: 10.1016/j.psj.2026.106575 (PMC12919266; doi:10.1016/j.psj.2026.106575)
Supplement: Supplementary file 1 [file mmc1.docx]

**Supplementary Table S2.** Seventeen representative isolates were selected for the detection of resistance genes by whole-genome sequencing.

| **No.** | **Farm** | **Isolate no.** | **Species** | **Year** | **Location** | **Age (Days)** | **Serotype** | **AMX** | **AMP** | **CTX** | **GEN** | **STR** | **DOX** | **TET** | **CHL** | **COL** | **SMX** | **TMP** |
| --- | --- | --- | --- | --- | --- | --- | --- | --- | --- | --- | --- | --- | --- | --- | --- | --- | --- | --- |
| 1 | H | RA15 | Layer duck | 2021 | Suphanburi | 28 | 7 | 4 | 32 | 0.03 | 32 | 16 | 1 | 4 | 2 | >128 | 64 | 0.5 |
| 2 | H | RA22 | Layer duck | 2021 | Suphanburi | 28 | 7 | 32 | 2 | 0.03 | 1 | 4 | 1 | 2 | 2 | >128 | 16 | 0.5 |
| 3 | D | RA54 | layer duck | 2022 | Nakhon Pathom | 47 | 11 | 4 | 1 | 0.25 | 128 | 128 | 1 | 1 | 2 | >128 | 128 | 128 |
| 4 | F | RA63 | broiler duck | 2022 | Nakhon Pathom |  | untypable | 4 | 4 | 0.03 | 64 | 32 | 2 | 8 | 2 | >128 | 128 | 128 |
| 5 | I | RA68 | layer duck | 2022 | Suphanburi | 40 | 1 | 16 | 8 | 0.06 | 32 | 16 | 2 | 16 | 2 | >128 | 128 | 128 |
| 6 | H | RA75 | broiler duck | 2022 | Suphanburi | 60 | 5 | 4 | 4 | 0.06 | 16 | 16 | 2 | 8 | 2 | >128 | 128 | 16 |
| 7 | E | RA79 | layer duck | 2023 | Nakhon pathom |  | 17 | 8 | 4 | 0.06 | 32 | 32 | 1 | 4 | 16 | 128 | 128 | 128 |
| 8 | C | RA85 | broiler duck | 2023 | Nakhon pathom | 25 | 10 | 4 | 2 | 0.03 | 64 | 32 | 2 | 16 | 2 | >128 | 128 | 64 |
| 9 | G | RA92 | Layer duck | 2023 | Nakhon pathom | 2-3 mo | 7 | 4 | 2 | 0.06 | 32 | 16 | 16 | 16 | 2 | >128 | 128 | 128 |
| 10 | C | RA95 | broiler duck | 2023 | Nakhon pathom | 4 wk | 7 | 4 | 2 | 0.06 | 32 | 16 | 1 | 4 | 2 | >128 | 128 | 8 |
| 11 | C | RA96 | broiler duck | 2023 | Nakhon pathom | 5 wk | untypable | 4 | 0.5 | 0.06 | 64 | 32 | 2 | 4 | 2 | >128 | 128 | 8 |
| 12 | C | RA99 | Breeder ducks | 2023 | Nakhon pathom | 4 wk | untypable | 4 | 2 | 0.03 | 64 | 32 | 2 | 8 | 2 | >128 | 128 | 32 |
| 13 | J | RA25 | layer chicken | 2022 | Kanchanaburi | 10 wks | 1 | 64 | 32 | 0.03 | 32 | 16 | 1 | 2 | 2 | >128 | 128 | 32 |
| 14 | J | RA27 | layer chicken | 2022 | Kanchanaburi | 10 wk | 1 | 2 | 32 | 0.03 | 8 | 64 | 1 | 2 | 0.5 | >128 | 64 | 32 |
| 15 | J | RA31 | layer chicken | 2022 | Kanchanaburi | 5 wk | 1 | 32 | 32 | 0.03 | 4 | 32 | 1 | 2 | 2 | >128 | 128 | 32 |
| 16 | J | RA34 | layer chicken | 2022 | Kanchanaburi | 5 wk | 1 | 0.5 | 2 | 0.06 | 32 | 32 | 0.25 | 2 | 2 | >128 | 128 | 16 |
| 17 | J | RA56 | layer chicken | 2022 | Kanchanaburi | 11 wk | 1 | 16 | 1 | 0.03 | 64 | 16 | 0.5 | 8 | 2 | >128 | 128 | 16 |
