# Supplementary material for: Genetic diversity, serotype, and antimicrobial profiles of Riemerella anatipestifer isolated from ducks and chickens in Thailand
Source: Poult Sci. 2026 Feb 2;105(4):106575. doi: 10.1016/j.psj.2026.106575 (PMC12919266; doi:10.1016/j.psj.2026.106575)
Supplement: Supplementary file 2 [file mmc2.docx]

**Supplementary Table S2** Profiles of antimicrobial resistance genes and their copy numbers

| **Resistance Gene** | **RA15** | **RA22** | **RA25** | **RA27** | **RA31** | **RA34** | **RA54** | **RA56** | **RA63** | **RA68** | **RA75** | **RA79** | **RA85** | **RA92** | **RA95** | **RA96** | **RA99** |
| --- | --- | --- | --- | --- | --- | --- | --- | --- | --- | --- | --- | --- | --- | --- | --- | --- | --- |
| *tet(*X2) | 1.78 | 1 | 0.96 | 1.01 | 0.93 | 0.93 | 1.48 | 0.98 | 0.99 | 0.97 | 0.9 | 0.9 | 2.78 | 0.88 | 0.87 | 0.97 | 0.97 |
| *bla*_RASA-1_ | 0.95 | - | - | - | - | - | 2.95 | - | 0.99 | 0.97 | 1.5 | - | - | 0.93 | 0.97 | - | - |
| *lnu*(I) | 0.95 | 0.89 | 0.91 | 0.94 | 0.99 | 0.99 | 1.53 | 1.01 | 0.99 | 0.97 | 1.71 | - | 1.9 | 0.93 | 0.97 | - | - |
| *ere*(D) | - | 0.89 | 0.91 | 0.94 | 0.99 | 0.99 | - | 1.01 | - | - | 0.99 | - | 1.9 | - | - | - | - |
| estT | 0.95 | - | - | - | - | - | 1.53 | - | - | - | 1.78 | 0.9 | - | - | - | - | - |
| *bla*_RAD-1_ | - | - | - | - | - | - | 0.71 | - | - | - | - | - | - | - | - | - | - |
| *erm*(F) | - | - | - | - | - | - | - | - | - | - | 0.88 | - | - | - | - | - | - |
| *lnu*(AN2) | - | - | - | - | - | - | - | - | - | - | - | 0.96 | - | - | - | - | - |
| *mef*(En2) | - | - | - | - | - | - | - | - | - | - | - | 0.96 | - | - | - | - | - |
| *tet*(Q) | - | - | - | - | - | - | - | - | - | - | - | 0.89 | - | - | - | - | - |
| *aadS* | - | - | - | - | - | - | - | - | - | - | - | 0.9 | - | - | - | - | - |
